# High initial IgG antibody levels against *Orientia tsutsugamushi* are associated with an increased risk of severe scrub typhus infection

**DOI:** 10.1371/journal.pntd.0009283

**Published:** 2021-03-18

**Authors:** Carol S. Devamani, John A. J. Prakash, Neal Alexander, William Stone, Karthik Gunasekaran, Winsley Rose, Wolf-Peter Schmidt

**Affiliations:** 1 Department of Clinical Microbiology, Christian Medical College, Vellore, India; 2 MRC Tropical Epidemiology Group, London School of Hygiene and Tropical Medicine, London, United Kingdom; 3 Department of Immunology and Infection, London School of Hygiene and Tropical Medicine, London, United Kingdom; 4 Department of Medicine, Christian Medical College, Vellore, India; 5 Department of Pediatrics and Pediatric Infectious Diseases, Christian Medical College, Vellore, India; 6 Department for Disease Control, London School of Hygiene and Tropical Medicine, London, United Kingdom; Seoul National University College of Medicine, REPUBLIC OF KOREA

## Abstract

**Background:**

Scrub typhus is a dominant cause of febrile illness in many parts of Asia. Immunity is limited by the great strain diversity of *Orientia tsutsugamushi*. It is unclear whether previous infection protects from severe infection or enhances the risk.

**Methods/principal findings:**

We studied IgG antibody levels against *O*. *tsutsugamushi* at presentation in 636 scrub typhus patients using enzyme-linked immunosorbent assays (ELISA). The association between ELISA optical density (OD) and risk of severe infection was modelled using Poisson regression. OD was categorised as low (<1.0), intermediate (1.0 to 2.9), and high (≥3.0). OD was also modelled as a continuous variable (cubic spline). Median age of cases was 41 years (range 0–85), with 37% having severe infection. Compared to the low category, the age-adjusted risk of severe infection was 1.5 times higher in the intermediate category (95%CI 1.2, 1.9), and 1.3 times higher in the high category (95%CI 1.0, 1.7). The effect was stronger in cases <40 years, doubling the risk in the intermediate and high categories compared to the low category. The effect was more pronounced in cases tested within 7 days of fever onset when IgG ODs are more likely to reflect pre-infection levels.

**Conclusions/Significance:**

Intermediate and high IgG antibody levels at the time of diagnosis are associated with a higher risk of severe scrub typhus infection. The findings may be explained by severe infection eliciting an accelerated IgG response or by previous scrub typhus infection enhancing the severity of subsequent episodes.

## Author summary

In some viral infections, repeat infection is associated with a higher risk of severe disease than the first infection. Clear clinical and epidemiological evidence for this effect only exists for dengue virus infection, but it may also play a role in non-viral infections. Scrub typhus is caused by intracellular bacteria of the *Orientia* genus, and is among the most common causes of febrile illness in many parts of Asia. Despite being common and potentially fatal, it remains a neglected and under-diagnosed infection. Due to the great strain diversity of *Orientia* species (especially *O*. *tsutsugamushi*) people can experience multiple infections during life. In this study of 636 scrub typhus cases from South India, we found elevated initial IgG antibody levels, possibly from an earlier infection, to be strongly associated with severe disease. The effect was especially pronounced in younger patients and at moderately elevated antibody levels. The findings may be explained by severe infection eliciting an accelerated IgG response or, as in dengue fever, by previous scrub typhus infection enhancing the risk of severe subsequent episodes.

## Introduction

Scrub typhus is a potentially life-threatening febrile illness caused by bacterial species belonging to the genus *Orientia* (family *Rickettsiaceae*) [1]. The infection is transmitted by the larvae (chiggers) of trombiculid mites [2]. Scrub typhus occurs over much of tropical and subtropical Asia as well as Chile [3]. In many endemic areas, scrub typhus accounts for 15% to 40% of febrile illness leading to hospitalisations [4–6]. Scrub typhus mortality has been estimated at 6% to 10% of untreated cases [2,7], which has been reduced by the use of antibiotics such as doxycycline or azithromycin [8,9]. Common manifestations of severe infection include acute respiratory distress syndrome (ARDS), meningo-encephalitis, shock and renal failure [4,9].

**Fig 1:**
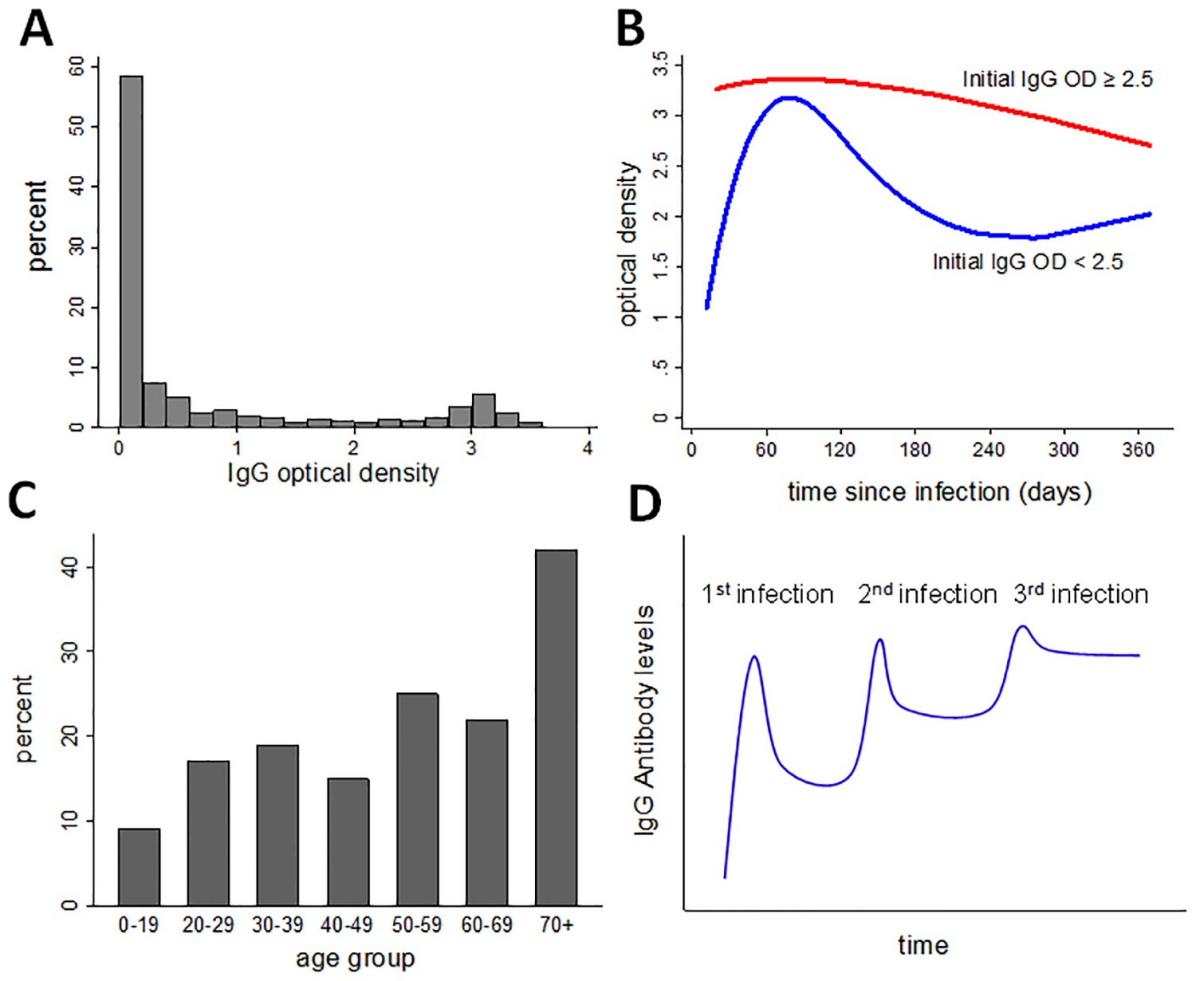
Sero-epidemiology of scrub typhus: A) IgG antibody levels expressed as ELISA optical density (OD) from cross-sectional study in the general population in the study setting (from [12]); B) IgG antibody response after infection in cases with high initial IgG antibody levels (red) and low initial IgG antibody levels (blue), from [14]; C) IgG sero-prevalence by age (from [11]); D) hypothesised building-up of IgG antibody levels over multiple scrub typhus infections.

Infection induces poor cross-protection between heterologous *O*. *tsutsugamushi* strains, the most common among the *Orientia* species [10]. Given the great strain diversity of *O*. *tsutsugamushi*, repeated infection with a variety of genetic variants may be a common phenomenon, possibly explaining some recent findings regarding the sero-epidemiology of scrub typhus. In cross-sectional surveys conducted in endemic areas in south India, IgG antibody levels against *O*. *tsutsugamushi* show a bimodal distribution with peaks at very low and very high levels (the latter are often regarded as “sero-positive”) [11–13] (Fig 1A). This is in contrast to *Rickettsia typhi* and the spotted fever group *Rickettsiae* against which IgG antibody responses do not show a second peak at higher values in the general population [12]. We have previously shown that the IgG response to scrub typhus infection in those with low initial IgG antibody levels was less pronounced and persistent compared to those with initially high IgG levels [14] (Fig 1B). This, together with the strong increase in IgG sero-positivity with age typically found in scrub typhus [11,12] (Fig 1C) suggests a gradual accumulation of IgG antibodies after multiple infections (Fig 1D). Similar dynamics have long been known for other infections, in particular Influenza [15].

The association between severity of illness and antibody response in infections that do not confer lasting immunity such as scrub typhus, malaria, influenza or dengue fever is complex. Severe infection may lead to a more pronounced humoral immune response and IgM/IgG antibody levels. Antibodies from an earlier infection, in particular IgG, may reduce the severity of subsequent infection of the same pathogen, as in malaria, or enhance severity as in dengue fever. Early reports of outbreaks in previously unexposed military personnel may suggest primary scrub typhus infection to be particularly severe [16,17] (reviewed by Elliott and colleagues [3]). However, re-infection leading to symptomatic [18] and severe infection [14] has been observed to occur within two years.

We previously found some evidence that cases of severe scrub typhus infection may on average have higher initial IgG antibody levels on initial presentation than uncomplicated cases [14]. Assuming that high initial IgG antibody levels reflect IgG antibody levels due to prior infections, we hypothesised that pre-existing antibodies to *O*. *tsutsugamushi* may increase the risk of severe infection. Due to a small sample size in our previous study [14] we were unable to explore whether the observed effect was directly associated with IgG antibody levels or due to confounding by age. Older individuals are at a higher risk of severe scrub typhus [4] and are also known to have higher levels of scrub typhus IgG (Fig 1C) [11,12]. We therefore retrospectively enrolled additional scrub typhus cases to investigate the association between initial IgG antibody levels and severe infection.

## Methods

### Ethics statement

The study was approved by CMC’s Institutional Review Board (CMC IRB Ref: 11726) and LSHTM’s Research Ethics Committee (LSHTM Ethics Ref: 16573). For prospectively enrolled cases, written consent was obtained from all adult participants. Written assent was obtained from minors, alongside written consent from their parents/guardians. All retrospectively enrolled cases (or their relatives) agreed to their data and samples collected for diagnosis and treatment to be used for research purposes.

### Study design, enrolment

This cross-sectional clinical study was conducted at the Christian Medical College Vellore (CMC), a tertiary care centre in the Indian state of Tamil Nadu. We aimed to enrol individuals diagnosed with scrub typhus and treated as in- or outpatients. We retrospectively enrolled cases with available blood sample (n = 470), in addition to cases prospectively enrolled for an earlier serological cohort study (n = 166) [14]. The 470 cases enrolled retrospectively had been treated at CMC for scrub typhus infection between September 2018 and March 2020. The prospective study had enrolled patients from two hospitals: 1) Patients of all ages treated at CMC (n = 126). Enrolment occurred in two phases, between September 2015 and February 2016 (cases <15 years), and between December 2017 and February 2019 (all ages). 2) Patients of all ages treated at a secondary care centre about 40km away from the main CMC hospital (n = 40). For this second group, enrolment occurred between August 2018 and February 2019. The total sample comprised 636 cases (S1 Fig).

### Blood testing

Blood samples (serum or citrate) were usually taken on the day of admission or the morning after admission. Serum was separated from blood cells, divided into aliquots and stored at -70°C until testing. We used enzyme-linked immunosorbent assays (ELISA) to detect IgM and IgG antibodies to *Orientia tsutsugamushi* (Scrub Typhus Detect, InBios International, Inc., Seattle, WA, USA) following the manufacturer’s specifications. The ELISA uses Karp, Kato, Gilliam and TA716 recombinant proteins of the 56-kD outer membrane protein. A phylogenetic analysis of *O*. *tsutsugamushi* isolates in Vellore showed that 65% were related to the Kato group, 31% to the Karp group and 4% to the Gilliam prototype [19]. The InBios ELISA has been shown to have sensitivities and specificities of over 90% in a study from Thailand [20], and 94% sensitivity and 92% specificity in a study from Vellore [21]. All assays were performed using an automated ELISA analyser (Euroimmun Analyzer1, Euroimmun, Lübeck, Germany) following the protocol of the Department of Clinical Microbiology which is an ISO15189:2012 accredited diagnostic laboratory. Serum samples were diluted 1:100 with the sample diluents. The absorbance was read at a wavelength of 450 nm. A patient was treated as a confirmed case of scrub typhus based on a single IgM ELISA OD value of 1.0 or higher in the absence of an alternative, plausible cause of fever. This cut-off has been locally defined for the diagnosis of acute cases in routine clinical practice and validated using the receiver operating characteristic approach in a sample of 346 subjects (scrub typhus cases, other fever cases, healthy controls) [22]. Forty-two patients were excluded despite a positive IgM test due to an unclear or alternative diagnosis (S1 Fig).

### Collection of clinical data

Clinical data were extracted from existing records. Severe scrub typhus infection was defined as the presence of at least one of the following clinical manifestations:

- Lung involvement–oxygen saturation below 92% and tachypnea at any time during admission. Tachypnea was defined as >20 breaths/minute in adults; children: >30/min aged 2–5 years, >25/min 5–12 years [23,24].
- Shock–adults: documented hypotension (systolic <90mmHg) at presentation or during treatment not responding to a single fluid bolus, or any documented use of inotropes. Children: documented hypotension <80mmHg (2–5 years), <90mmHg (5–12 years), or any documented use of inotropes; or capillary refill time >2s with tachycardia >150/min (2–5 years), >130/min (5–12 years) [23,24].
- Kidney injury–any creatinine of 3.0 mg/dl or higher in the absence of a known, pre-existing chronic kidney disease.
- Central nervous system (CNS)–any focal neurological deficit, or any elevated white blood cell counts in a cerebrospinal fluid sample, or any focal or generalised seizure in an adult, or any focal or generalised seizure in a child not diagnosed as simple febrile seizure. Simple febrile seizure in children less than 6 years of age was assumed if there was no more than one generalised seizure lasting less than 15 minutes.
- Myocarditis: New onset heart failure confirmed by echocardiography with an elevated troponin T in a patient with no known heart condition.
- Large vessel occlusion, eg. peripheral gangrene or organ infarct.
- Severe bleeding manifestation: purpura fulminans, gastro-intestinal and urinary tract haemorrhage.

The researcher extracting the clinical data (CD) was blind to the IgG status of cases.

### Statistical analysis

All analyses were done in STATA 14. The risk ratios for the association between the IgG ELISA OD and the risk of severe infection were calculated using modified Poisson regression with robust standard errors according to Zou [25]. The effect of age and the duration of fever until blood testing on IgG OD was modelled using quantile regression [26]. Models were done separately for the 33^rd^, 50^th^ (median), and 67^th^ percentiles. To allow for non-linearity, age and duration of fever were modelled as restricted cubic splines, with the location of knots chosen following Harrell [27], and their number restricted to 4.

**Fig 2:**
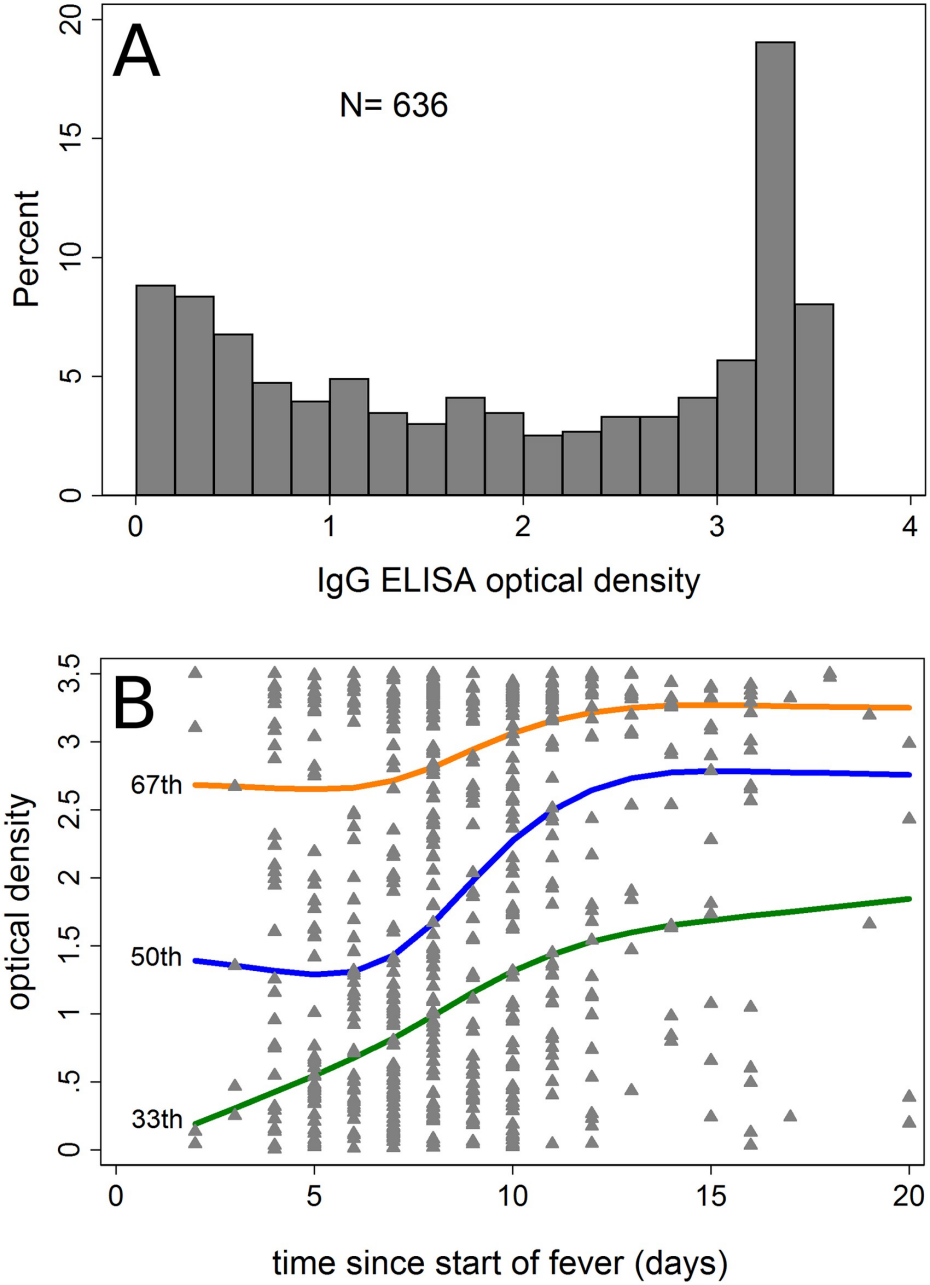
A) Histogram of IgG optical density values at presentation. B) Association between duration of fever until scrub typhus test and IgG optical density. Displayed are the 33th, 50^th^ (median) and 67^th^ percentiles predicted using cubic spline models (4 knots). Triangles show individual values.

OD values of the IgG class were categorised as <1.0, 1.0 to <3.0 and 3.0 or above, conforming to the bimodal distribution of values (Fig 2A). In further analysis, we modelled OD values using restricted cubic splines to avoid the need for cut-off points. The number of knots was restricted to 5, with the position of knots chosen following Harrell [27]. To address confounding by age, models were adjusted for age as a quadratic term. We explored whether disease severity affected the findings by restricting the inclusion of severe cases to those with lung involvement (as the most common life-threatening organ involvement [4]) and to those with at least two organs involved (Table 1). We explored effect modification of the association between OD and risk of severe infection by age (<40 years vs ≥40 years) using likelihood ratio tests. We conducted further subgroup analyses by duration of fever prior to testing (≤7 days vs >7days), and presence of an eschar vs no eschar. We again used median regression (i.e. quantile regression based on the 50^th^ percentile) to compare median OD values of the IgM class between severe and non-severe scrub typhus cases, and between the three categories of OD for the IgG class. As missing data were rare, no imputation was done.

**Table 1:** Characteristics of cases.

	n/N	% or mean (SD, range)
**Total**	636/636	100
**Female gender** (2 missing values)	337/634	53
**Age group** (years)		
0–9	113/636	18
10–19	49/636	8
20–29	66/636	10
30–39	77/636	12
40–49	89/636	14
50–59	127/636	20
≥60	115/636	18
**Duration of fever prior to scrub typhus test** (days, 6 missing values)	-	9.0 (4.2, 2–37)
**Eschar present** (2 missing values)	302/634	48
**Organ involvement**		
Lungs	146/636	23
Shock	92/636	14
Kidney injury	47/636	7
CNS	58/636	8
Myocarditis	13/636	2
Gastro-intestinal bleed	4/636	0.6
Digital gangrene	2/636	0.3
Other*	4/636	0.6
**Died during admission**	20/636	3
**Any organ involvement**	235/636	37
**Number of organs involved**		
0	401	63
1	139	22
2	60	9
3	24	4
≥4	12	2
**Respiratory support**		
None	469/636	74
Oxygen mask / nasal prongs	50/636	8
Non-invasive ventilation	65/636	10
Invasive ventilation	52/636	8

* One case each of secondary hemophagocytic lymphohistiocytosis, bowel ischemia, splenic infarct and haematuria.

## Results

Characteristics of cases are shown in Table 1. Females and individuals aged below 10 years and above 50 predominated. The mean age was 37 years (median 41.5). The mean duration of fever prior to the scrub typhus test was 9 days (median 8). In approximately half of cases an eschar was found. Lung involvement was the most common clinical manifestation of severe infection, followed by shock, CNS and kidney injury. Fifteen percent of cases had more than one organ involvement, while 3% of cases died. Respiratory support was given in a quarter of cases with 8% requiring invasive ventilation.

Gender was not strongly associated with the risk of severe infection (Table 2). There was some evidence for an increased risk of severe infection in the age groups 10 to 29 years and above 50 years of age. There was no clear trend towards a longer duration of fever prior to testing being associated with severe infection. By contrast, the presence of an eschar was associated with a 40% increased risk.

**Table 2:** Factors associated with the risk of severe infection.

	N	Crude risk	RR	95%CI
**Total**	636	37.0%	-	-
**Gender**				
Male	297	39.1%	1.0 (ref)	-
Female	337	35.0%	0.9	0.7, 1.1
**Age**				
0–9	113	28.3%	1.0 (ref)	
10–19	49	38.8%	1.4	0.9, 2.2
20–29	66	39.4%	1.4	0.9, 2.1
30–39	77	26.0%	0.9	0.6, 1.5
40–49	89	34.8%	1.2	0.8, 1.9
50–59	127	39.4%	1.4	1.0, 2.0
≥60	115	49.6%	1.8	1.2, 2.5
**Duration of fever until testing**				
≤6 days	150	40.7%	1.0 (ref)	
7 to 10 days	316	33.9%	0.8	0.7, 1.1
≥11 days	164	40.2%	1.0	0.8, 1.3
**Eschar**				
Absent	332	31.6%	1.0 (ref)	
Present	302	42.7%	1.4	1.1, 1.7

The histogram of IgG ELISA optical densities of cases showed a strongly bimodal distribution (Fig 2A), similar to what is known from cross-sectional surveys in the area (Fig 1A). Thirty-three percent of cases had an IgG OD below 1.0, 34% between 1.0 and <3.0 and 33% 3.0 or above. Both the 33^rd^ and the 67^th^ fitted OD percentiles showed a gradual increase with longer duration of fever (Fig 2B). For the median percentile, there was initially a gradual increase with an accelerated increase after day 7. There was a gradual increase in IgG with age for the 50^th^ and 67^th^ percentiles (S2 Fig).

In crude analysis, cases in the intermediate OD category (≥1.0 to <3.0) had a 40% higher risk of severe infection than those with an IgG OD below 1.0, which increased to 50% after adjusting for age (Table 3). Cases in the highest OD category (≥3.0) tended to have a higher risk of severe infection than those in the lowest category but a lower risk than those in the intermediate category.

**Table 3:** Effect of IgG optical density on the risk of severe infection with subgroup analysis by age and duration of fever at time of testing.

	N	Crude risk	RR	95%CI	RR*	95%CI	Test for inter-action
*All cases*							
<1.0	207	29.5%	1.0 (ref)	-	1.0 (ref)		
1.0 to <3.0	218	42.7%	1.4	1.1, 1.9	1.5	1.2, 1.9	
≥3.0	211	38.4%	1.3	1.0, 1.7	1.3	1.0, 1.7	
*Restricted to cases with lung involvement*							
<1.0	185	21.0	1.0 (ref)		1.0 (ref)		
1.0 to <3.0	187	31.2	1.6	1.1, 2.2	1.6	1.2, 2.3	
≥3.0	175	25.7	1.2	0.8, 1.8	1.1	0.8, 1.7	
*Restricted to cases without lung involvement*							
<1.0	168	13.1	1.0 (ref)		1.0 (ref)		
1.0 to <3.0	156	19.9	1.5	0.9, 2.5	1.5	0.9, 2.5	
≥3.0	166	21.7	1.7	1.0, 2.7	1.7	1.0, 2.7	
*Restricted to cases with 2 or more organs involved*							
<1.0	176	17.1	1.0 (ref)		1.0 (ref)		
1.0 to <3.0	168	25.6	1.5	1.0, 2.3	1.6	1.1, 2.4	
≥3.0	153	15.0	0.9	0.5, 1.5	0.8	0.5, 1.4	
*Restricted to cases with only one organ involved*							
<1.0	177	17.5	1.0 (ref)		1.0 (ref)		
1.0 to <3.0	175	28.6	1.6	1.1, 2.4	1.6	1.1, 2.4	
≥3.0	188	30.9	1.8	1.2, 2.6	1.7	1.2, 2.5	
*Subgroup analysis by age*							0.07
**<40 years**							
<1.0	101	18.8%	1.0 (ref)		1.0 (ref)		
1.0 to <3.0	121	39.7%	2.1	1.3, 3.3	2.1	1.3, 3.3	
≥3.0	83	36.1%	1.9	1.2, 3.2	1.8	1.1, 3.0	
**≥ 40 years**							
<1.0	106	39.6%	1.0 (ref)		1.0 (ref)		
1.0 to <3.0	97	46.4%	1.2	0.9, 1.6	1.2	0.9, 1.6	
≥3.0	128	39.8%	1.0	0.7, 1.4	1.0	0.7, 1.4	
*Subgroup analysis by duration of fever until testing*							0.44
**≤ 7 days**							
<1.0	99	28.3%	1.0 (ref)	-	1.0 (ref)		
1.0 to <3.0	71	43.7%	1.5	1.0, 2.3	1.6	1.1, 2.4	
≥3.0	60	45.0%	1.6	1.0, 2.4	1.5	1.0, 2.3	
**> 7 days**							
<1.0	108	30.6%	1.0 (ref)		1.0 (ref)		
1.0 to <3.0	147	42.2%	1.4	1.0, 1.9	1.4	1.0, 2.0	
≥3.0	151	35.8%	1.2	0.8, 1.7	1.1	0.8, 1.6	
*Subgroup analysis by presence of an eschar*							0.465
Eschar present							
<1.0	100	33.0%	1.0 (ref)		1.0 (ref)		
1.0 to <3.0	112	46.4%	1.4	1.0, 2.0	1.4	1.0, 2.0	
≥3.0	90	48.9%	1.5	1.0, 2.1	1.4	1.0, 2.0	
Eschar absent							
<1.0	107	26.1%	1.0 (ref)		1.0 (ref)		
1.0 to <3.0	105	38.1%	1.5	1.0, 2.2	1.4	1.0, 2.1	
≥3.0	120	30.8%	1.2	0.8, 1.8	1.1	0.8, 1.7	

* adjusted for age as quadratic term

When restricting the analysis to cases with lung involvement (excluding cases without lung involvement), cases in the intermediate OD category were still those with the highest risk of severe infection (Table 3), while the risk of severe infection for cases in the highest OD category was quite similar to the risk in the lowest OD category. The same was found in the analysis restricted to cases with at least two organs involved (excluding cases with only one organ involvement). By contrast, in the analyses excluding cases with lung involvement or those with two or more organs involved, there was little difference in the risk of severe infection between the intermediate and highest OD category (Table 3).

Age appeared to strongly modify the association between IgG OD and the risk of severe infection (Table 3). In those aged less than 40 years, the risk of severe infection was twice as high in the intermediate category and about 80 to 90% higher in the high category compared to the lowest category. In those aged 40 years or older, the association was only weak (test for interaction: p = 0.07).

Because of higher IgG OD values in cases presenting later for testing (Fig 2B), we conducted a subgroup analysis in those where the ELISA test was done within 7 days of fever onset vs those in whom the test was done later. In those tested within 7 days, the difference in risk between the lowest and the other two categories was higher than in those tested after 7 days of fever (Table 3). Overall, the effect of initially intermediate or high IgG levels on the risk of severe infection appeared attenuated in those tested after 7 days of fever, although there was no strong evidence for interaction. Presence or absence of an eschar did not modify the association between OD and the risk of severe infection.

**Fig 3:**
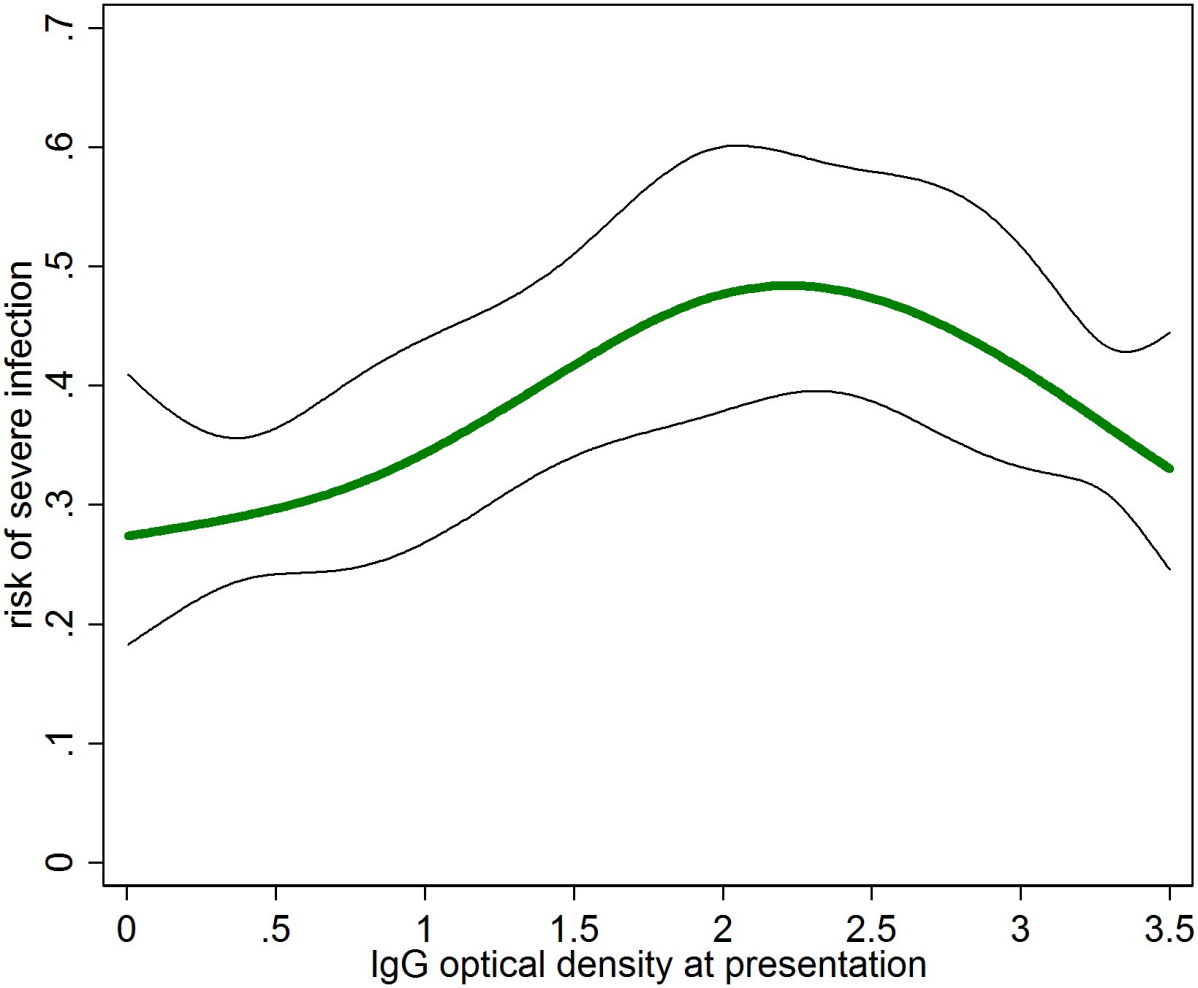
Association between IgG optical density (OD) and the risk of severe infection with 95% CI. OD modelled as cubic splines with 5 knots.

Fig 3 shows the association between IgG OD and the risk of severe infection calculated using cubic splines with 5 knots, avoiding the need for defining OD cut-off values. The overall analysis confirmed a particularly high risk of cases with intermediate IgG OD values (Fig 3). The subgroup analysis by age confirmed a pronounced effect of IgG OD on the risk of severe infection in those aged less than 40 years while the effect was attenuated in those aged 40 or older but not quite to the extent as observed in the less flexible categorical analysis. In particular, the peak risk was shifted to lower OD values compared to the younger ages. In subgroup analysis by duration of fever, the peak in the risk of severe infection was slightly shifted towards higher OD values in those presenting after 7 days of fever.

Median IgM OD values differed little between severe (3.13) and non-severe scrub typhus cases (3.07, difference in medians 0.06, 95%CI -0.07, 0.20). Median OD values of the IgM class were 2.75 in the category defined by low values for the IgG class, 3.14 in the intermediate category and 3.15 in the high category. Thus, compared to the low IgG category, both the intermediate and the high IgG OD categories had about 0.38 (95%CI 0.25, 0.52) and 0.39 (95%CI 0.26, 0.53) higher median IgM OD values.

## Discussion

In this analysis of more than 600 scrub typhus cases from South India, intermediate and high initial IgG antibody levels at presentation were associated with a markedly higher risk of severe infection compared to cases presenting with initially low IgG antibody levels. There was evidence that this effect was particularly pronounced in those aged less than 40 years.

**Fig 4:**
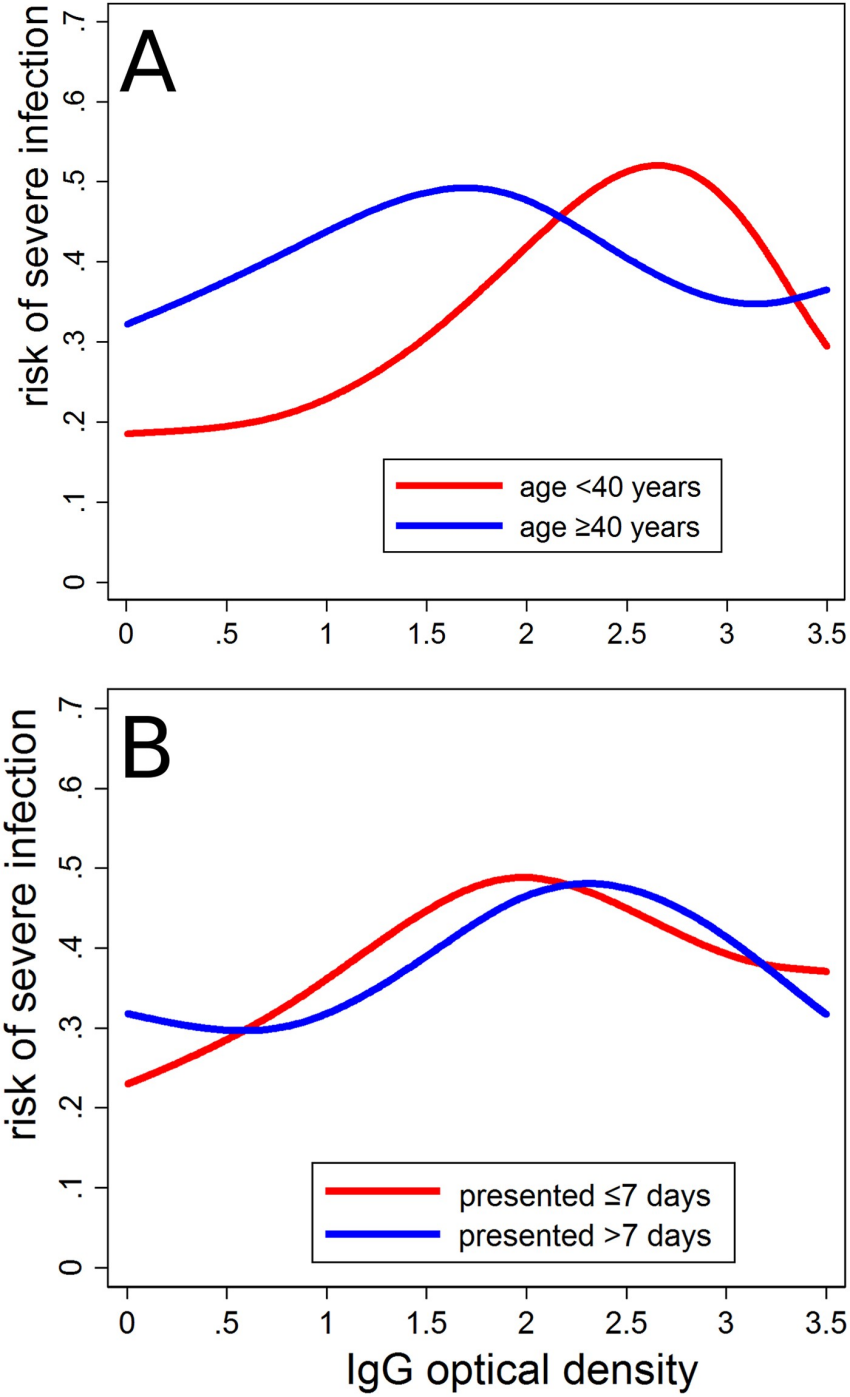
Association between IgG optical density (OD) and the risk of severe infection stratified by (A) age and (B) duration of fever until testing. OD modelled as cubic splines with 5 knots.

If IgG antibodies to *O*. *tsutsugamushi* in the early phase of illness largely reflect pre-infection levels, then our findings may suggest that a scrub typhus infection in the past may increase the risk of severe infection in subsequent episodes. We believe there is some indication for initial antibody levels to predominantly reflect pre-infection levels: First, the bimodal distribution found in the initial IgG values in this study and the increase of IgG levels with age (S2 Fig) were similar to what has been found in cross sectional surveys of asymptomatic people in the general population in the same setting (Fig 1A and 1C), making it unlikely that high IgG values were due only to the current infection. Second, as with many other infections, IgG levels for scrub typhus rise gradually over weeks after infection, reaching a peak only at about 3 months [14]. In the present study, we similarly found IgG levels to increase only slowly during the first 7 days of fever (Fig 2B), suggesting that during this time IgG levels may only be marginally higher than prior to the infection. The effect of initial IgG levels on the risk of severe infection was more pronounced in cases tested within these 7 days than in those tested later (Table 3 and Fig 4). If there is a true effect of prior IgG levels on the risk of severe infection then one would expect this effect to be attenuated in those tested later, as their antibody levels may reflect prior levels less closely. One would further expect the peak of the effect to shift to higher OD values in those tested later, as IgG is likely to increase in most infected regardless of the starting point. Both observations were made in this study (Table 3 and Fig 2B). The fitted median IgG OD levels underwent an accelerated growth from day 7, possibly suggesting activation of memory cells from an earlier infection (the lack of accelerated growth in the 67^th^ percentile is likely due to test limitations at high values). We therefore believe that the data are compatible with prior scrub typhus infection enhancing the severity of subsequent infection. In dengue fever, prior infection is known to cause more severe subsequent infections, through a mechanism called antibody-dependent enhancement. Whether scrub typhus infection is modified by antibody-dependent enhancement or other mechanisms enhancing subsequent episodes has not been systematically studied. Antibody-dependent enhancement is of concern in the development of dengue fever vaccines, some of which have been shown to enhance subsequent infection by mimicking primary infection, demonstrating the public health importance of this effect [28].

Despite these considerations, the absence of data on pre-infection IgG levels in the cases included in this study makes it difficult to exclude the possibility of reverse causality as the main limitation of this study. High bacterial loads in severe cases may accelerate IgG response even in those who have never been infected with *O*. *tsutsugamushi* before, which would lead to an observed association between IgG levels and risk of severe infection.

The study is further limited by the way most cases in this study were diagnosed, relying on a single cut-off for IgM ELISA in the absence of an alternative plausible diagnosis. Since scrub typhus is the leading cause of hospitalisations for undifferentiated fever in this setting [4,11], the positive predictive value of a single positive IgM ELISA is likely to be high, given the high estimated specificity of this test (>90%) in South India [21,29]. Further, the analysis was unaffected by excluding cases without eschar (Table 3). Presence of an eschar alone or in combination with a positive ELISA is likely to be highly specific for scrub typhus in this setting [30], especially since in this region, eschars are rare in spotted fever [31].

Effective antimicrobials such as doxycycline and azithromycin were given to all patients in this study. It is unclear how many of the uncomplicated infections included here would have become severe without treatment, making the clinical study of severe scrub typhus more difficult than that of severe dengue fever, where treatment is largely supportive. In this study, duration of fever until testing was not associated with the risk of severe infection, in contrast to a study from Shandong, China [8].

The findings are further limited by lack of data on which strains of *O*. *tsutsugamushi* caused scrub typhus in this study. Variations in strain diversity across settings, and the sequence of strains causing primary infection and re-infection may influence the risk of severe infection [32] and perhaps the way in which one infection may enhance subsequent infection. The genotypes identified by an earlier study in the same setting as the present analysis were closely related to the Karp-, Kato- and Gilliam prototype strains [19] and appear to be well matched by the recombinant antigens ELISA antigens used here. However, data on the locally circulating serotypes which may better reflect the suitability of the ELISA used are not available. Further, differences in the affinity of antibodies elicited by various strains to ELISA antigens may have impacted the OD values used in this analysis. Moreover, the relationship between OD values and actual antibody concentrations is not straightforward. However, it is not clear whether the use of semi-quantitative methods such as endpoint titration using indirect immunofluorescence assays would have offered any advantages over an ELISA test based on recombinant antigens [33].

As the increased risk of severe infection in those with intermediate and high initial IgG levels was robust to age adjustment, and was particularly pronounced in younger age groups, confounding by age does not seem to be a likely explanation for the findings. However, age groups may differ in the likelihood of seeking healthcare. The cases enrolled in this study may not represent the full spectrum of scrub typhus infection in this area.

Despite these limitations, we believe that the effect sizes observed in this study are large enough to merit further study. These may include in vitro studies, for example by using cell culture. Clinical studies exploring IgG antibody levels in mothers of very young scrub typhus cases may be used to estimate the effect of maternal antibodies on the severity of infection in the child. Such studies could contribute to ongoing work to identify determinants of scrub typhus severity in general.

## Supporting information

S1 Fig Study flow diagram. (TIF)

S2 Fig Association between age and IgG optical density. Displayed are the 33th, 50^th^ (median) and 67^th^ percentiles predicted using cubic spline models (4 knots). Triangles show individual values.
(TIF)

S1 Text STROBE statement. (DOCX)
