# Supplementary figures and images for: High initial IgG antibody levels against Orientia tsutsugamushi are associated with an increased risk of severe scrub typhus infection
Source: PLoS Negl Trop Dis. 2021 Mar 18;15(3):e0009283. doi: 10.1371/journal.pntd.0009283 (PMC8009433; doi:10.1371/journal.pntd.0009283)

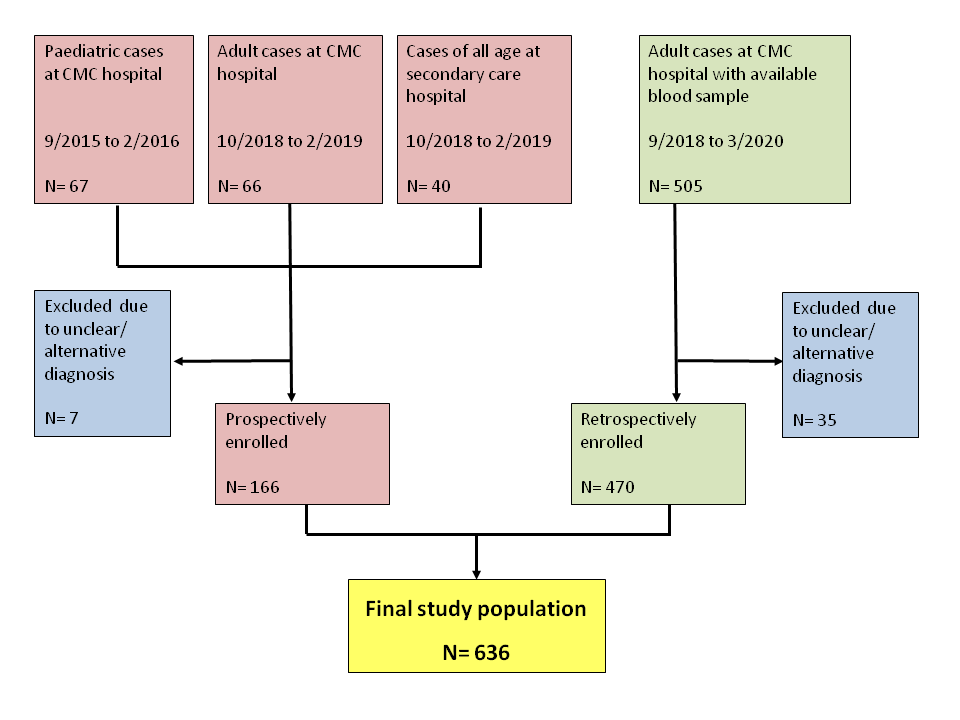

Supplement: S1 Fig — (TIF) [file pntd.0009283.s001.tif]

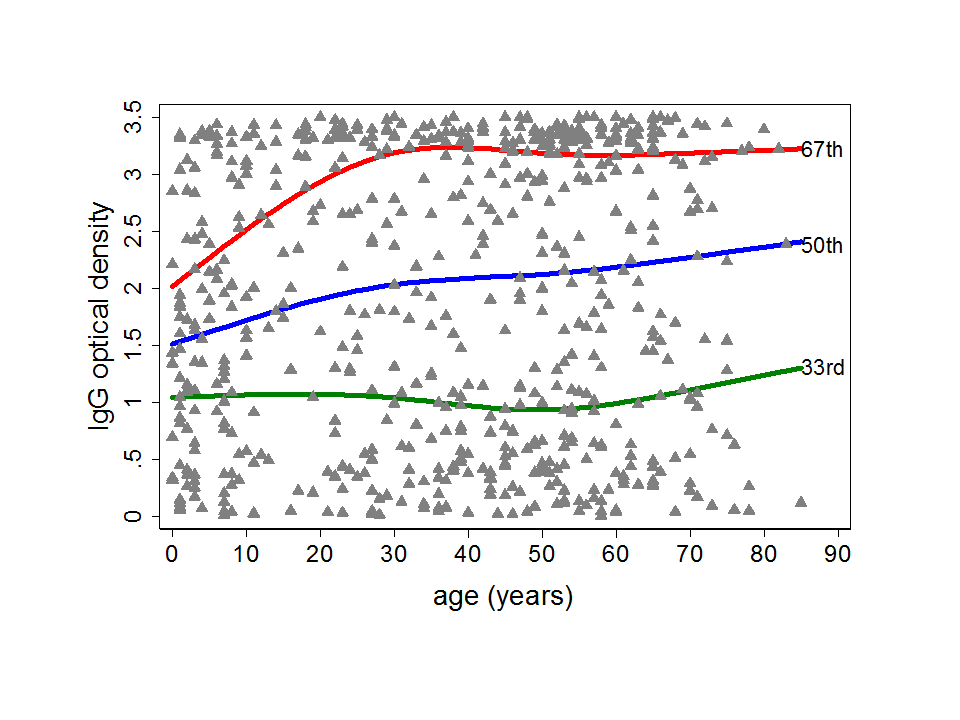

Supplement: S2 Fig — Displayed are the 33th, 50th (median) and 67th percentiles predicted using cubic spline models (4 knots). Triangles show individual values. (TIF) [file pntd.0009283.s002.tif]
